# Perinatal outcomes of twin pregnancies complicated by early twin‐to‐twin transfusion syndrome treated with fetoscopic laser surgery

**DOI:** 10.1002/uog.70196

**Published:** 2026-03-30

**Authors:** F. G. Sileo, A. Khalil, J. Binder, E. Brunelli, N. Chianchiano, C. M. Coutinho, F. D'Antonio, M. Döbert, A. Fichera, Y. Gielchinsky, K. Hecher, C. Iacovella, S. Malone, A. Martinez‐Varea, L. N. Nørgaard, C. Rodo, T. Simões, F. Slaghekke, Y. Yinon, Collaborators, F. Bahlmann, E. Carreras, S. Gueli Alletti, O. Yaghi, E. Lopriore, M. M. Okido, A. Markovich, D. Mohammed, E. Moreno, S. Prasad, F. Prefumo, A. Queirós, J. Morales‐Roselló, K. Sundberg, M. Yeoh, A. Youssef

**Affiliations:** ^1^ Fetal Medicine Unit, St George's University Hospitals NHS Foundation Trust University of London London UK; ^2^ Prenatal Medicine Unit University of Modena and Reggio Emilia Modena Italy; ^3^ Vascular Biology Research Centre Molecular and Clinical Sciences Research Institute, St George's University of London London UK; ^4^ Department of Obstetrics and Feto‐Maternal Medicine Medical University of Vienna Vienna Austria; ^5^ Obstetric Unit, Department of Medical and Surgical Sciences University of Bologna and IRCCS Azienda Ospedaliero‐Universitaria S.Orsola‐Malpighi Bologna Italy; ^6^ Fetal Medicine Unit, Bucchieri La Ferla‐Fatebenefratelli Hospital Palermo Italy; ^7^ Hospital das Clínicas da Faculdade de Medicina de Ribeirão Preto da Universidade de São Paulo Ribeirão Preto Brazil; ^8^ Center for Fetal Care and High‐Risk Pregnancy, Department of Obstetrics and Gynecology University “G. d'Annunzio” of Chieti‐Pescara Chieti Italy; ^9^ Department of Obstetrics and Fetal Medicine University Medical Center Hamburg‐Eppendorf Hamburg Germany; ^10^ Department of Clinical and Experimental Sciences University of Brescia Brescia Italy; ^11^ Fetal Medicine Center, Helen Schneider Hospital for Women, Rabin Medical Center Petach Tikvah Israel; ^12^ Faculty of Medical and Health Sciences Tel Aviv University Tel Aviv Israel; ^13^ Department of Obstetrics and Gynecology Dr. Senckenbergische Stiftung, Buergerhospital Frankfurt am Main Germany; ^14^ Department of Maternal‐Fetal Medicine Royal Women's Hospital Melbourne Australia; ^15^ Department of Obstetrics and Gynecology La Fe University and Polytechnic Hospital Valencia Spain; ^16^ Center of Fetal Medicine, Department of Obstetrics Copenhagen University Hospital Rigshospitalet Copenhagen Denmark; ^17^ Department of Obstetrics and Reproductive Medicine Maternal‐Fetal Medicine Unit, Grup de Recerca en Medicina Materna I Fetal, Vall d'Hebron Institut de Recerca (VHIR), Vall d'Hebron Hospital Universitari Barcelona Spain; ^18^ Department of Maternal‐Fetal Medicine and Maternity Dr. Alfredo da Costa Nova Medica School Lisbon Portugal; ^19^ Department of Obstetrics and Feto‐Maternal Medicine Leiden University Medical Center Leiden The Netherlands; ^20^ Gray Faculty of Medical and Health Sciences Tel‐Aviv University Tel Aviv Israel

**Keywords:** early, intrauterine demise, laser, MCDA, monochorionic diamniotic, survival, TTTS, twin‐to‐twin transfusion syndrome

## Abstract

**Objective:**

The aim of this study was to evaluate the perinatal outcomes of monochorionic diamniotic (MCDA) twin pregnancies complicated by early twin‐to‐twin transfusion syndrome (TTTS) treated with fetoscopic laser surgery (FLS), and to compare rates of fetal survival across Quintero stages.

**Methods:**

This was a multicenter retrospective cohort study of MCDA pregnancies complicated by TTTS diagnosed ≤ 18 weeks' gestation (early TTTS) that underwent FLS from January 2007 to August 2023. Monoamniotic twins, triplets or higher‐order multiple gestations, and pregnancies complicated by genetic or structural anomalies were excluded. Demographic data, gestational age at diagnosis and at FLS, Quintero stage at diagnosis and details of management of the pregnancy, including complications and perinatal outcomes, were obtained from patient records at each participating center. The primary outcome was survival at 28 days after birth.

**Results:**

In total, 678 MCDA pregnancies with early TTTS were identified, of which 550 underwent FLS. Of these, 485 cases had a known pregnancy outcome and were therefore included. The median gestational age at diagnosis was 17 + 0 (interquartile range (IQR), 16 + 3 to 17 + 4) weeks, and median gestational age at FLS was 17 + 4 (IQR, 17 + 0 to 17 + 6) weeks. The most common Quintero stage at diagnosis was Stage III (46.0% (223/485)). At least one postoperative complication occurred in 36.5% (177/485) of cases, including preterm prelabor rupture of membranes, intrauterine fetal demise of the cotwin, vaginal bleeding, septostomy and pregnancy loss. Dual‐twin survival was reported in 51.5% (250/485) of cases, while survival of at least one twin was reported in 76.7% (372/485). There were no surviving fetuses in 23.3% (113/485) of pregnancies. When considering cases diagnosed ≤ 16 weeks, the rate of survival of at least one twin was 87.5% for cases diagnosed at Quintero Stages I–II *vs* 59.5% for Stages III–IV (*P* = 0.019).

**Conclusions:**

The diagnosis and management of TTTS ≤ 18 weeks are more complex than those of TTTS diagnosed > 18 weeks. More early‐TTTS cases were diagnosed at Quintero Stage III, but the overall survival rate was not significantly different between cases diagnosed at Quintero Stages I–II *vs* Stages III–IV, except when the diagnosis was made ≤ 16 weeks, in which case the survival rate was higher among cases diagnosed at Stages I–II. Revisions to the diagnostic criteria for early TTTS should be considered. © 2026 The Author(s). *Ultrasound in Obstetrics & Gynecology* published by John Wiley & Sons Ltd on behalf of International Society of Ultrasound in Obstetrics and Gynecology.

## INTRODUCTION

Twin‐to‐twin transfusion syndrome (TTTS) is one of the most severe complications of monochorionic diamniotic (MCDA) twin pregnancies, with an estimated incidence of 10–15%^1^, ^2^. Timely diagnosis and treatment of TTTS are crucial for improving perinatal outcomes. Prenatal intervention options include fetoscopic laser surgery (FLS) of the placental anastomoses, which is more effective than serial amnioreduction before 26 weeks' gestation^3^. Other management modalities include expectant management and selective fetal reduction using techniques such as cord occlusion. The latter might be chosen to reduce the risk of mortality and severe morbidity, including neurological injuries due to a single fetal death, or when the chance of survival of both twins is extremely low^4^, ^5^. Improvements in surgical expertise, technical equipment and image quality, in addition to neonatal care, have led to a significant reduction in perinatal mortality and neurological morbidity in cases of TTTS^6^, ^7^, ^8^, ^9^, ^10^.

TTTS most commonly develops after 18 weeks' gestation. However, a subset of cases may develop at an earlier gestational age (GA) and are defined accordingly as ‘early TTTS’. These cases, despite being rare, may also require treatment before 18 weeks^11^, although the evidence on management, prognosis and survival rates for early TTTS is mainly derived from relatively small observational studies on this specific subgroup^12^. FLS is conventionally performed between 18 and 26 weeks, although some studies have shown that it can be performed ≤ 18 weeks^13^, ^14^, ^15^. However, it is unclear if FLS performed ≤ 18 weeks is associated with worse pregnancy outcomes compared with FLS performed > 18 weeks, owing to technical challenges and/or a higher stage of TTTS^16^, ^17^. We hypothesized that the outcomes of FLS for TTTS ≤ 18 weeks are similar to those > 18 weeks.

At present, there are no robust data on the actual risk of perinatal mortality and morbidity or on the optimal management of early TTTS according to GA at presentation, limiting the ability to provide individualized management and counseling. The primary aims of this multicenter study were to report the perinatal outcomes of MCDA twin pregnancies complicated by early TTTS and treated with FLS of the placental anastomoses, regardless of the GA at which FLS was performed, and to compare rates of fetal survival across Quintero stages according to the GA at diagnosis and at FLS. Secondary aims were to describe and establish the rate of early and late postoperative complications, such as preterm prelabor rupture of membranes (PPROM), intrauterine fetal demise (IUFD) and recurrent TTTS.

## METHODS

### Study design and population

This was a retrospective multicenter cohort study analyzing prospectively collected data on MCDA twin pregnancies undergoing FLS for early TTTS (i.e. diagnosed ≤ 18 + 0 weeks' gestation) from January 2007 to August 2023. All participating centers were tertiary fetal medicine referral centers providing care for complex monochorionic pregnancies; Table S1 provides a list of the participating centers. The inclusion criteria were MCDA pregnancies complicated by TTTS diagnosed ≤ 18 + 0 weeks and treated with FLS of the placental anastomoses. Exclusion criteria were triplet and higher‐order multiple pregnancies, monochorionic monoamniotic twin pregnancies, MCDA pregnancies complicated by structural or chromosomal anomaly and cases of early TTTS not treated with FLS of the placental anastomoses. Patients underwent a first‐trimester scan at 11 + 0 to 13 + 6 weeks in order to establish GA and chorionicity, to label the twins and to screen for aneuploidy according to local guidelines. Afterwards, from 16 weeks or earlier according to local protocols, patients received a scan at least every 2 weeks.

GA was determined according to the crown–rump length of the larger fetus in pregnancies conceived spontaneously, or according to the date of oocyte retrieval or embryonic age from fertilization in pregnancies conceived via *in‐vitro* fertilization. Monochorionicity was determined by the presence of a thin intertwin membrane at the site of insertion of the amniotic membrane into the placenta (the T‐sign)^18^. Options for twin labeling included identification of each twin based on its position relative to the maternal cervix, its intrauterine location (right *vs* left or lower *vs* upper) and/or the site of umbilical‐cord insertion relative to the placental margins and intertwin membrane. Each center followed its own protocol for crown–rump length or nuchal translucency (NT) discordance as possible early predictors of TTTS^19^, ^20^ in terms of additional scans performed between the first‐trimester scan and the 16‐week scan. At each of these scans, the estimated fetal weight (EFW) and the deepest vertical pocket (DVP) of amniotic fluid were assessed for each fetus. Screening for aneuploidy, detailed anatomical ultrasound scans and monitoring for monochorionicity‐related complications were performed as per local or international guidelines^18^. As part of the evaluation of early TTTS, an anomaly scan was performed before and after FLS to identify any brain or cardiac anomalies. Data on the placental pathological examination were not included in this study.

A diagnosis of TTTS was made if polyhydramnios–oligohydramnios sequence (i.e. DVP ≥ 8 cm in recipient twin and ≤ 2 cm in donor twin) was observed, and was classified according to Quintero staging^21^. However, even in the absence of polyhydramnios, cases were considered to have TTTS if there was amniotic fluid volume (AFV) discordance, defined as any obvious intertwin AFV difference not fulfilling the conventional criteria for TTTS^22^; a distended bladder in the recipient twin, defined as a large bladder with rapid filling, and an absent bladder in the donor twin^23^; and/or signs of cardiac dysfunction, consisting of cardiomegaly, cardiac hypertrophy, tricuspid regurgitation or a reversed ‘a’ wave of the ductus venosus (DV) on Doppler ultrasound^24^. A diagnosis of twin anemia–polycythemia sequence (TAPS) was made when there was intertwin discordance in peak systolic velocity (PSV) of the middle cerebral artery (MCA) of more than 1 multiple of the median (MoM) or when MCA‐PSV was > 1.5 MoM in the donor twin and < 0.8 MoM in the recipient twin^25^, ^26^.

Selective fetal growth restriction (sFGR) was defined as either EFW < 10^th^ centile in one twin combined with an intertwin EFW discordance ≥ 25%, or EFW < 3^rd^ centile in one twin^18^, ^27^. Intertwin EFW discordance was calculated using the formula: ((EFW of the larger fetus – EFW of the smaller fetus) / EFW of the larger fetus) × 100.

### Study outcomes and data collection

Data on maternal age, mode of conception, GA at diagnosis of TTTS, Quintero stage at diagnosis, coexisting sFGR and fetal Doppler parameters at diagnosis were extracted from patient records. Data on the management of TTTS after diagnosis were collected, including selective fetal reduction, FLS or any active fetal intervention (including amniodrainage) or expectant management. If selective fetal reduction or another intervention was performed, data on the indication, GA at intervention and technique were recorded. In cases managed with FLS (i.e. those included in this study), data on complications (e.g. vaginal bleeding, PPROM within 7 days after FLS, single IUFD within 7 days after FLS, pregnancy loss (defined as double IUFD or miscarriage) within 7 days after FLS, recurrence of TTTS and the diagnosis of TAPS and/or sFGR) were also retrieved. Perinatal outcomes, including live birth, IUFD, neonatal death within 28 days after birth and termination of pregnancy (TOP), were documented. Data on GA at birth were also collected when available. The primary outcome was survival at 28 days after birth.

### Data acquisition

A standardized Microsoft Excel 365 spreadsheet (Microsoft Corp., Redmond, WA, USA) was used to collect all the available information listed above for each pregnancy separately. The secure database of each center was searched, and the retrieved data were anonymized and recorded on the Excel spreadsheet independently in each center. Pregnancy outcomes were obtained from the maternity database and neonatal records. The anonymized data were transferred securely to the study coordinator, who analyzed the outcomes and stored them on an encrypted hospital hard drive. Ethical approval and data governance procedures were followed at all participating centers in accordance with local regulations. The coordinating center (St George's Hospital, London, UK) confirmed that retrospective analysis of routinely collected anonymous data does not require formal ethical approval.

### Statistical analysis

Continuous variables are presented as median (interquartile range (IQR)) or mean ± SD. Normality assumptions were tested using the Shapiro–Wilk test. Continuous variables were compared using either a *t*‐test or the Wilcoxon rank‐sum test. Categorical variables are presented as *n* (%) and were compared using the chi‐square test or Fisher's exact test as appropriate. *P* < 0.05 was considered statistically significant. All analyses were carried out using SPSS version 24 (IBM Corp., Armonk, NY, USA).

## RESULTS

### Study cohort selection

**Figure 1:**
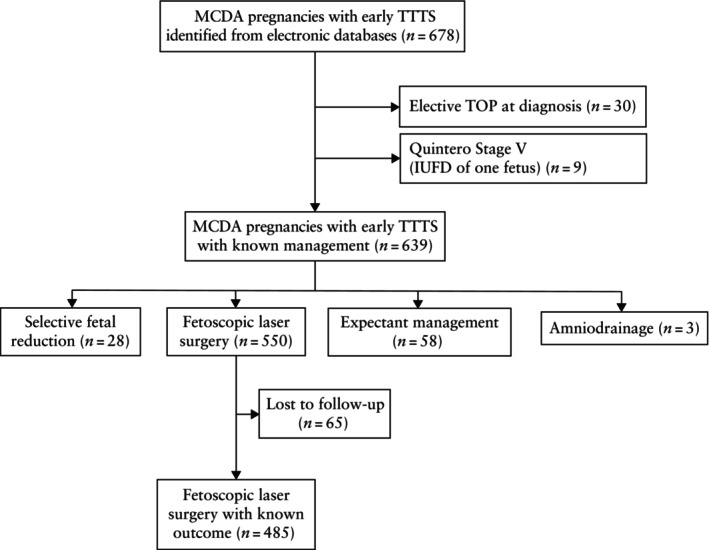
Flowchart showing inclusion in study of monochorionic diamniotic (MCDA) twin pregnancies diagnosed with early twin‐to‐twin transfusion syndrome (TTTS) and treated with fetoscopic laser surgery. IUFD, intrauterine fetal demise; TOP, termination of pregnancy.

During the study period, 678 women with early TTTS were identified from the electronic databases of the participating hospitals. Of the entire cohort, 4.4% (30/678) of women opted for TOP after the diagnosis of early TTTS (Figure 1). Nine cases presented with Quintero Stage‐V TTTS (i.e. IUFD of one fetus) at diagnosis and were excluded from further analysis. After excluding pregnancies managed expectantly, those that underwent amniodrainage only or selective fetal reduction and those lost to follow‐up, the study cohort included 485 MCDA pregnancies treated with FLS for early TTTS with a known outcome. The general characteristics of the study cohort are presented in Table 1.

**Table 1:** General characteristics of 485 monochorionic diamniotic twin pregnancies diagnosed with early twin‐to‐twin transfusion syndrome and treated with fetoscopic laser surgery (FLS)

Characteristic	Value
Maternal age (years)	31.2 ± 5.2
Mode of conception	
Spontaneous	388/440 (88.2)
ART	52/440 (11.8)
Gestational age at diagnosis (weeks)	17 + 0 (16 + 3 to 17 + 4)
Quintero stage at diagnosis	
Stage I	55 (11.3)
Stage II	187 (38.6)
Stage III	223 (46.0)
Stage IV	20 (4.1)
Gestational age at FLS (weeks)*	17.5 ± 1.0
Concomitant selective fetal growth restriction	130 (26.8)

Data are given as mean ± SD, *n*/*N* (%), median (interquartile range) or *n* (%).

* Information available for 480/485 cases. ART, assisted reproductive technology.

### Fetoscopic laser surgery

Of the 485 pregnancies that underwent FLS, the diagnosis of TTTS occurred at a median GA of 17 + 0 (IQR, 16 + 3 to 17 + 4) weeks and the mean GA at FLS was 17.5 ± 1.0 weeks. Most cases presented with Quintero Stage III (46.0% (223/485)) or Stage II (38.6% (187/485)) at diagnosis (Table 1). FLS was performed ≤ 72 h after diagnosis in 338/480 (70.4%) cases, > 72 h and ≤ 1 week after diagnosis in 43/480 (9.0%) and > 1 week after diagnosis in 99/480 (20.6%). There was no information available on the GA at or timing of FLS in five (1.0%) patients. The characteristics of the cohort according to the time interval from diagnosis to FLS are presented in Table 2.

**Table 2:** Interval between diagnosis of early twin‐to‐twin transfusion syndrome in monochorionic diamniotic twin pregnancies and fetoscopic laser surgery (FLS), according to Quintero stage and gestational age (GA) at diagnosis

	GA at diagnosis (weeks)			
Timing of FLS	≤ 15 + 0	15 + 1 to 16 + 0	16 + 1 to 17 + 0	17 + 1 to 18 + 0
≤ 72 h after diagnosis	1/338 (0.3)	23/338 (6.8)	128/338 (37.9)	186/338 (55.0)
Stage I	0/17 (0)	0/17 (0)	10/17 (58.8)	7/17 (41.2)
Stage II	0/127 (0)	8/127 (6.3)	44/127 (34.6)	75/127 (59.1)
Stage III	0/180 (0)	12/180 (6.7)	69/180 (38.3)	99/180 (55.0)
Stage IV	1/14 (7.1)	3/14 (21.4)	5/14 (35.7)	5/14 (35.7)
> 72 h and ≤ 1 week after diagnosis	0/43 (0)	9/43 (20.9)	24/43 (55.8)	10/43 (23.3)
Stage I	0/8 (0)	0/8 (0)	3/8 (37.5)	5/8 (62.5)
Stage II	0/19 (0)	3/19 (15.8)	11/19 (57.9)	5/19 (26.3)
Stage III	0/15 (0)	5/15 (33.3)	10/15 (66.7)	0/15 (0)
Stage IV	0/1 (0)	1/1 (100)	0/1 (0)	0/1 (0)
> 1 week after diagnosis	8/99 (8.1)	20/99 (20.2)	32/99 (32.3)	39/99 (39.4)
Stage I	0/28 (0)	2/28 (7.1)	11/28 (39.3)	15/28 (53.6)
Stage II	2/39 (5.1)	9/39 (23.1)	12/39 (30.8)	16/39 (41.0)
Stage III	5/28 (17.9)	9/28 (32.1)	8/28 (28.6)	6/28 (21.4)
Stage IV	1/4 (25.0)	0/4 (0)	1/4 (25.0)	2/4 (50.0)
No information on timing	0/5 (0)	0/5 (0)	1/5 (20.0)	4/5 (80.0)

Data are given as *n*/*N* (%).

Early complications (≤ 7 days after FLS) and late complications (> 7 days after FLS) were recorded. At least one complication was recorded in 177/485 (36.5%) cases. In terms of early complications, PPROM was recorded in 13.8% (67/485) of cases (of which FLS was performed < 17 + 0 weeks in 23/67), single IUFD in 19.0% (92/485), pregnancy loss in 7.0% (34/485), vaginal bleeding in 3.5% (17/485) and septostomy in 3.1% (15/485). In terms of late complications, recurrent TTTS occurred in 4.3% (21/485), with repeat FLS performed in 1.2% (6/485). Post‐FLS TAPS was observed in 6.4% (31/485) of cases. Early and late complications (according to GA and Quintero stage at diagnosis) are reported in Tables S2a and S2b, respectively.

Table 3 reports early complications according to GA at FLS < 18 weeks or ≥ 18 weeks, showing significantly different rates of pregnancy loss and vaginal bleeding between the two groups, but no significant difference in rates of PPROM, single IUFD or septostomy.

**Table 3:** Early complications (≤ 7 days after fetoscopic laser surgery (FLS)) of monochorionic diamniotic pregnancies with early twin‐to‐twin transfusion syndrome, according to gestational age at FLS

Early complication	FLS < 18 weeks (*n* = 390)	FLS ≥ 18 weeks (*n* = 90)	*P*
PPROM	58 (14.9)	9 (10.0)	0.115
Single IUFD	71 (18.2)	20 (22.2)	0.648
Septostomy	12 (3.1)	2 (2.2)	0.664
Vaginal bleeding	17 (4.4)	0 (0)	0.044
Pregnancy loss	32 (8.2)	2 (2.2)	0.046

Data are presented as *n* (%). IUFD, intrauterine fetal demise; PPROM, preterm prelabor rupture of membranes.

Table 4 presents the perinatal outcomes of pregnancies with early TTTS treated with FLS, according to GA and Quintero stage at diagnosis. Overall, 51.5% (250/485) of pregnancies had dual‐twin survival, 25.2% (122/485) had single‐twin survival and 23.3% (113/485) had dual‐twin demise.

**Table 4:** Survival outcomes of monochorionic diamniotic pregnancies with early twin‐to‐twin transfusion syndrome treated with fetoscopic laser surgery, according to gestational age (GA) and Quintero stage at diagnosis

GA at diagnosis	Dual‐twin demise	Single‐twin survival	Dual‐twin survival
≤ 16 + 0 weeks	18/61 (29.5)	15/61 (24.6)	28/61 (45.9)
Stage I	0/2 (0)	1/2 (50.0)	1/2 (50.0)
Stage II	3/22 (13.6)	5/22 (22.7)	14/22 (63.6)
Stage III	13/31 (41.9)	8/31 (25.8)	10/31 (32.3)
Stage IV	2/6 (33.3)	1/6 (16.7)	3/6 (50.0)
16 + 1 to 17 + 0 weeks	38/185 (20.5)	47/185 (25.4)	100/185 (54.1)
Stage I	4/25 (16.0)	7/25 (28.0)	14/25 (56.0)
Stage II	12/67 (17.9)	19/67 (28.4)	36/67 (53.7)
Stage III	21/87 (24.1)	19/87 (21.8)	47/87 (54.0)
Stage IV	1/6 (16.7)	2/6 (33.3)	3/6 (50.0)
17 + 1 to 18 + 0 weeks	57/239 (23.8)	60/239 (25.1)	122/239 (51.0)
Stage I	3/28 (10.7)	4/28 (14.3)	21/28 (75.0)
Stage II	26/98 (26.5)	23/98 (23.5)	49/98 (50.0)
Stage III	24/105 (22.9)	31/105 (29.5)	50/105 (47.6)
Stage IV	4/8 (50.0)	2/8 (25.0)	2/8 (25.0)

Data are given as *n*/*N* (%).

When stratifying by GA at diagnosis, FLS was performed ≤ 72 h after diagnosis in 39.3% (24/61) of cases diagnosed ≤ 16 + 0 weeks, 69.6% (128/184) of those diagnosed between 16 + 1 and 17 + 0 weeks and 79.1% (186/235) of those diagnosed > 17 + 0 weeks. FLS was performed > 72 h and ≤ 1 week after diagnosis in 14.8% (9/61) of the women diagnosed ≤ 16 + 0 weeks, 13.0% (24/184) of those diagnosed between 16 + 1 and 17 + 0 weeks and 4.3% (10/235) of those diagnosed > 17 + 0 weeks. FLS was performed > 1 week after diagnosis in the remaining 45.9% (28/61) of the women diagnosed ≤ 16 + 0 weeks, 17.4% (32/184) of those diagnosed between 16 + 1 and 17 + 0 weeks and 16.6% (39/235) of those diagnosed > 17 + 0 weeks. Table S3 presents the perinatal outcomes of early TTTS cases treated with FLS, categorized by Quintero stage and GA at diagnosis and the interval between TTTS diagnosis and FLS.

After excluding pregnancies with no information on the timing of FLS (5/485 (1.0%)) and those with no survivors (113/480 (23.5%)), among the remaining 76.5% (367/480) of pregnancies with at least one survivor, GA at birth was recorded in 355 pregnancies. Among these, preterm birth < 28 + 0 weeks occurred in 11.0% (39/355) of pregnancies, while only 10.7% (38/355) of births occurred at term. Table 5 presents the data on GA at birth according to the timing of FLS and GA at diagnosis. The median interval between FLS and delivery in this group was 15.7 (IQR, 12.9–18.0) weeks.

**Table 5:** Gestational age (GA) at birth in 355 monochorionic diamniotic pregnancies with early twin‐to‐twin transfusion syndrome treated with fetoscopic laser surgery (FLS) that had at least one surviving twin, according to GA at diagnosis and timing of FLS

	GA at birth (weeks)				
GA at diagnosis/timing of FLS	< 28 + 0	28 + 0 to 31 + 6	32 + 0 to 33 + 6	34 + 0 to 36 + 6	≥ 37 + 0
All*	39/355 (11.0)	85/355 (23.9)	88/355 (24.8)	105/355 (29.6)	38/355 (10.7)
≤ 16 + 0	4/42 (9.5)	11/42 (26.2)	10/42 (23.8)	11/42 (26.2)	6/42 (14.3)
FLS ≤ 72 h	3/17 (17.6)	5/17 (29.4)	2/17 (11.8)	6/17 (35.3)	1/17 (5.9)
FLS > 72 h and ≤ 1 week	0/7 (0)	2/7 (28.6)	2/7 (28.6)	2/7 (28.6)	1/7 (14.3)
FLS > 1 week	1/18 (5.6)	4/18 (22.2)	6/18 (33.3)	3/18 (16.7)	4/18 (22.2)
16 + 1 to 17 + 0 weeks	16/144 (11.1)	35/144 (24.3)	33/144 (22.9)	51/144 (35.4)	9/144 (6.3)
FLS ≤ 72 h	12/96 (12.5)	20/96 (20.8)	22/96 (22.9)	35/96 (36.5)	7/96 (7.3)
FLS > 72 h and ≤ 1 week	2/21 (9.5)	6/21 (28.6)	6/21 (28.6)	7/21 (33.3)	0/21 (0)
FLS > 1 week	2/27 (7.4)	9/27 (33.3)	5/27 (18.5)	9/27 (33.3)	2/27 (7.4)
17 + 1 to 18 + 0 weeks	19/169 (11.2)	39/169 (23.1)	45/169 (26.6)	43/169 (25.4)	23/169 (13.6)
FLS ≤ 72 h	18/131 (13.7)	27/131 (20.6)	36/131 (27.5)	31/131 (23.7)	19/131 (14.5)
FLS > 72 h and ≤ 1 week	0/8 (0)	2/8 (25.0)	2/8 (25.0)	3/8 (37.5)	1/8 (12.5)
FLS > 1 week	1/30 (3.3)	10/30 (33.3)	7/30 (23.3)	9/30 (30.0)	3/30 (10.0)

Data are given as *n*/*N* (%).

* Total of pregnancies with information on GA at birth and at least one live birth.

### Survival rates according to Quintero stage, gestational age at diagnosis, timing of FLS and coexistence of sFGR

The rate of survival of at least one twin was significantly different (*P* = 0.0156) between Quintero stages for cases of early TTTS diagnosed ≤ 16 + 0 weeks (Table 6). When data were grouped to compare rates of at least one survivor between Stages I–II and Stages III–IV, the survival rate was significantly higher when the diagnosis was made at Stages I–II (87.5% *vs* 59.5%; *P* = 0.019). No significant differences in survival rate were found among those diagnosed between 16 + 1 and 17 + 0 weeks and those diagnosed > 17 + 0 weeks (*P* > 0.05), both across individual stages and when comparing Stages I–II with Stages III–IV (Table 6).

**Table 6:** Rate of survival of at least one twin in monochorionic diamniotic pregnancies with early twin‐to‐twin transfusion syndrome treated with fetoscopic laser surgery, according to gestational age (GA) at diagnosis and Quintero stage

GA at diagnosis	Stage I	Stage II	Stage III	Stage IV	*P*	Stages I–II	Stages III–IV	*P*
≤ 16 + 0 weeks	2/2 (100)	19/22 (86.4)	18/31 (58.1)	4/6 (66.7)	0.0156	21/24 (87.5)	22/37 (59.5)	0.019
16 + 1 to 17 + 0 weeks	21/25 (84.0)	55/67 (82.1)	66/87 (75.9)	5/6 (83.3)	0.7185	76/92 (82.6)	71/93 (76.3)	0.292
17 + 1 to 18 + 0 weeks	25/28 (89.3)	72/98 (73.5)	81/105 (77.1)	4/8 (50.0)	0.106	97/126 (77.0)	85/113 (75.2)	0.749

Data are given as *n*/*N* (%).

There was no significant difference in the rate of survival of at least one twin when cases were grouped according to Quintero Stages I–II and Stages III–IV and stratified by GA at FLS (≤ 16 + 0 weeks, between 16 + 1 weeks and 18 + 0 weeks and > 18 + 0 weeks) (Table S4). Additionally, there were no significant differences in the rate of survival of at least one twin in those with *vs* without coexisting sFGR in cases of Stages I–II (75.9% (41/54) *vs* 81.4% (153/188); *P* = 0.375) and Stages III–IV (71.1% (54/76) *vs* 74.3% (124/167); *P* = 0.601).

## DISCUSSION

Early TTTS, diagnosed ≤ 18 weeks' gestation, is an uncommon pregnancy complication with a reported incidence of approximately 2.5% of all TTTS cases^28^. This is the largest cohort study, reporting data on 485 MCDA twin pregnancies complicated by early TTTS and treated with FLS.

FLS is currently offered when TTTS is diagnosed ≤ 18 weeks, but owing to a lack of well‐defined diagnostic criteria or a consistent management strategy, it is probable that clinicians only offer FLS for more severe cases. This is supported by the fact that, in this cohort, Quintero Stage III was the most common stage at TTTS diagnosis (46% of cases), and even more common (50.8% (31/61)) among the cases diagnosed ≤ 16 weeks. Similar findings have been reported previously in a systematic review and meta‐analysis of Mustafa *et al*.^16^ and a recent cohort study of Seaman *et al*.^17^. The difficulty in diagnosing TTTS at an earlier GA appears to be, at least in part, owing to the fact that AFV discordance does not meet the classic criteria when the onset of TTTS occurs before 18 weeks. It has been suggested that, for the recipient twin, there is less urinary contribution to the AFV at an earlier GA, which limits the development of polyhydramnios. Meanwhile, for the donor twin, continued transudation from maternal plasma across the fetal skin and placental surfaces may limit the development of oligohydramnios^29^.

The overall mortality rate observed in our study is similar to that of previous reports on early TTTS. A systematic review of D'Antonio *et al*.^12^ reporting on perinatal outcomes in early TTTS, including 13 studies and 104 pregnancies, found a perinatal death rate of 47.3% in cases managed with FLS compared to 43.9% in cases managed expectantly. However, the small numbers in this study created some limitations, with a lack of standard criteria for antenatal surveillance and management and a lack of stratification for the stage of TTTS. Espinoza *et al*.^15^ recently compared outcomes of MCDA twin pregnancies complicated by early TTTS treated with FLS before and after 18 weeks, with 68 cases undergoing FLS < 18 weeks and a larger cohort of 346 cases undergoing FLS ≥ 18 weeks. They did not find a significant difference in the rates of extreme preterm delivery, PPROM or neonatal survival between the two groups. The mortality rate in the group treated < 18 weeks was 27.9%.

We noted a moderate increase in overall survival rate (survival of at least one twin) with advancing GA at diagnosis of TTTS, ranging from 70.5% in the group diagnosed ≤ 16 + 0 weeks to 76.2% in the group diagnosed between 17 + 1 and 18 + 0 weeks. The most significant difference in survival was seen in the group diagnosed ≤ 16 + 0 weeks when comparing Quintero Stages I–II (87.5%) with Stages III–IV (59.5%). This suggests that, with consistent criteria to allow for the earlier diagnosis of TTTS at lower Quintero stages, a significant improvement could be made to outcomes.

Current guidelines recommend the initiation of surveillance of MCDA pregnancies for TTTS at 16 weeks^18^. The ability to predict the development of TTTS at an earlier GA might support an earlier onset of surveillance and allow for diagnosis at a lower Quintero stage. NT discordance at the first‐trimester ultrasound has been suggested as a predictor for the development of TTTS. Kagan *et al*.^19^ evaluated 512 MCDA twins between 11 and 13 + 6 weeks. They showed that 52% of pregnancies with a NT discordance of ≥ 20% developed TTTS requiring FLS. However, the odds for prediction of TTTS only became significant when NT discordance was ≥ 30%. A regression analysis showed that the ability of discordance in NT to predict the development of TTTS was 1.06 (95% CI, 1.04–1.08) and 1.05 (95% CI, 1.03–1.07) for univariable and multivariable analyses, respectively. Mogra *et al*.^30^ found that the best predictive marker for TTTS was a NT discordance of ≥ 20%, with an area under the receiver‐operating‐characteristics curve of 0.79 (95% CI, 0.59–0.99) but a positive predictive value of only 36%. Additionally, a recent study of Thi *et al*.^31^ found that a NT discordance of > 20% was not associated with the development of TTTS. Thus, it would appear that NT discordance is of limited value in predicting the development of TTTS.

One suggestion to improve the diagnosis of early TTTS would be to modify the AFV discordance criteria^32^. Van Mieghem *et al*.^22^ reported that a moderate AFV discordance of > 3.1 cm before 20 weeks indicates a greater risk for the development of TTTS, with a sensitivity of 81.8% and a specificity of 43.9%. Hayashi *et al*.^33^ advocated for an AFV discordance of ≥ 4.0 cm to define a moderate difference in AFV between 16 and 26 weeks. While neither recommendation is specific to early GA, they can provide a framework for modifying the AFV discordance to be used as the diagnostic criterion for TTTS in pregnancies ≤ 18 weeks, thus allowing for an earlier diagnosis. If this degree of AFV discordance is not acceptable as an indication for intervention, it should be considered an indication for more frequent follow‐up to detect progression of TTTS.

Cardiac changes, such as ventricular hypertrophy, atrioventricular valve regurgitation and abnormalities in the DV waveform, have been noted more frequently in the recipient twin in more advanced stages of TTTS^34^. More detailed analysis of the DV waveforms has shown shorter diastolic velocity‐time integrals and filling times in the recipient twin compared with the donor^35^. However, neither of these studies focused on whether these changes may be useful in making a diagnosis of TTTS at an earlier stage (≤ 18 weeks) and cardiac changes could not be evaluated in the latter study owing to its retrospective design. Future research should focus on defining alternative diagnostic parameters, including cardiac changes, for cases in which the classic staging criteria cannot be met, so that appropriate prenatal therapy can be offered in a timely manner^36^.

In the absence of appropriate diagnostic criteria and owing to the technical challenges associated with interventions early in gestation, FLS may be delayed, particularly for early TTTS. Stirnemann *et al*.^7^ reported a 10% increase in the risk of PPROM within 1 week after the surgery when FLS was performed < 17 weeks compared with ≥ 17 weeks. The increased risk of PPROM may be related to the fact that the amnion and chorion fusion may be incomplete before 18 weeks. The rate of PPROM within 1 week of surgery in this cohort was 14.9% when FLS was performed < 18 weeks, compared with 9% when FLS was performed ≥ 18 weeks. While the difference is not statistically significant, clinicians may still be inclined to delay surgery until after 18 weeks.

Other technical challenges, including a lower AFV in the recipient sac or the smaller size of the uterus and placenta, may make visualization more challenging, particularly when there is an anterior placenta. Difficult visualization may lead to incomplete ablation of all anastomoses, increasing the risk of recurrent TTTS and TAPS. The use of larger endoscopes may also contribute to the incidence of PPROM, while using smaller‐diameter endoscopes makes visualization more challenging. The benefit of using FLS for Stage‐I TTTS in general remains unproven, and there are definite risks associated with the procedure^37^. The question remains whether this applies equally to early TTTS. Thus, even if the diagnostic criteria for TTTS are modified, there are still barriers to offering FLS to this group, which are the most likely to benefit in terms of improved survival.

### Strengths and limitations

The main strength of this study is the large number of MCDA twin gestations drawn from 17 different fetal centers. However, the retrospective nature of the study presents a limitation in terms of missing data and could introduce a risk of bias. The number of centers also creates a limitation in terms of heterogeneity in definitions used among the various centers, as well as in protocols for management and follow‐up. This could, however, also enhance the external validity of the study, as the results reflect real‐world experience. The broad time‐span during which cases were included may also be a limitation, as there were changes in surgical technique, equipment and neonatal care over this time period that could have influenced outcomes.

### Conclusions

Early TTTS is relatively rare, and its diagnosis and management are more complex than those of TTTS diagnosed > 18 weeks. A greater proportion of early‐TTTS cases were diagnosed at Quintero Stage III. However, even when FLS was performed, the overall survival rate was not significantly different between cases diagnosed at Quintero Stages I–II and those diagnosed at Stages III–IV, except when diagnosis occurred < 16 weeks, in which case survival was higher among Stages‐I–II TTTS. These findings suggest that revisions to the diagnostic criteria for early TTTS should be considered.

## Collaborators

**F. Bahlmann**, Department of Obstetrics and Gynecology, Dr. Senckenbergische Stiftung, Buergerhospital, Frankfurt am Main, Germany

**E. Carreras**, Department of Obstetrics and Reproductive Medicine, Maternal‐Fetal Medicine Unit, Grup de Recerca en Medicina Materna I Fetal, Vall d'Hebron Institut de Recerca (VHIR), Vall d'Hebron Hospital Universitari, Barcelona, Spain

**S. Gueli Alletti**, Fetal Medicine Unit, Bucchieri La Ferla‐Fatebenefratelli Hospital, Palermo, Italy

**O. Yaghi**, Fetal Medicine Unit, St George's University Hospitals NHS Foundation Trust, University of London, London, UK

**E. Lopriore**, Department of Pediatrics, Leiden University Medical Center, Leiden, The Netherlands

**M. M. Okido**, Hospital das Clínicas da Faculdade de Medicina de Ribeirão Preto da Universidade de São Paulo. Ribeirão Preto, Brazil

**A. Markovich**, Gray Faculty of Medical and Health Sciences, Tel‐Aviv University, Tel Aviv, Israel

**D. Mohammed**, Fetal Medicine Unit, St George's University Hospitals NHS Foundation Trust, University of London, London, UK

**E. Moreno**, Department of Obstetrics and Reproductive Medicine, Maternal‐Fetal Medicine Unit, Grup de Recerca en Medicina Materna I Fetal, Vall d'Hebron Institut de Recerca (VHIR), Vall d'Hebron Hospital Universitari, Barcelona, Spain

**S. Prasad**, Fetal Medicine Unit, St George's University Hospitals NHS Foundation Trust, University of London, London, UK

**F. Prefumo**, UOC Ostetricia e Ginecologia, IRCCS Istituto Giannina Gaslini, Genova, Italy

**A. Queirós**, Department of Maternal‐Fetal Medicine and Maternity Dr. Alfredo da Costa, Nova Medica School, Lisbon, Portugal

**J. Morales‐Roselló**, Department of Obstetrics and Gynecology, La Fe University and Polytechnic Hospital, Valencia, Spain

**K. Sundberg**, Center of Fetal Medicine, Department of Obstetrics, Copenhagen University Hospital, Rigshospitalet, Copenhagen, Denmark

**M. Yeoh**, Department of Maternal‐Fetal Medicine, Royal Women's Hospital, Melbourne, Australia

**A. Youssef**, Obstetric Unit, Department of Medical and Surgical Sciences, University of Bologna and IRCCS Azienda Ospedaliero‐Universitaria S. Orsola‐Malpighi, Bologna, Italy.

## Supporting information


**Table S1** List of participating centers.
**Table S2** Early complications within 7 days (a) and late complications (b) of cases of early twin‐to‐twin transfusion syndrome treated with fetoscopic laser surgery according to gestational age and Quintero stage at diagnosis.
**Table S3** Survival outcomes of cases of early twin‐to‐twin transfusion syndrome diagnosed ≤ 16 + 0 weeks (a), between 16 + 1 and 17 + 0 weeks (b) and between 17 + 1 and 18 + 0 weeks (c) treated with fetoscopic laser surgery (FLS) according to Quintero stage at diagnosis and timing of FLS from diagnosis.
**Table S4** Comparison of rates of survival of at least one twin in Quintero Stages I–II and Stages III–IV twin‐to‐twin transfusion syndrome cases treated with fetoscopic laser surgery according to gestational age (GA) at laser.

## Data Availability

The data that support the findings of this study are available on request from the corresponding author. The data are not publicly available due to privacy or ethical restrictions.
