# Supplementary material for: Perinatal outcomes of twin pregnancies complicated by early twin‐to‐twin transfusion syndrome treated with fetoscopic laser surgery
Source: Ultrasound Obstet Gynecol. 2026 Mar 30;67(4):461–9. doi: 10.1002/uog.70196 (PMC13040126; doi:10.1002/uog.70196)
Supplement: Supplementary file 1 — Table S1 List of participating centers. Table S2 Early complications within 7 days (a) and late complications (b) of cases of early twin‐to‐twin transfusion syndrome treated with fetoscopic laser surgery according to gestational age and Quintero stage at diagnosis. Table S3 Survival outcomes of cases of early twin‐to‐twin transfusion syndrome diagnosed ≤ 16 + 0 weeks (a), between 16 + 1 and 17 + 0 weeks (b) and between 17 + 1 and 18 + 0 weeks (c) treated with fetoscopic laser surgery (FLS) according to Quintero stage at diagnosis and timing of FLS from diagnosis. Table S4 Comparison of rates of survival of at least one twin in Quintero Stages I–II and Stages III–IV twin‐to‐twin transfusion syndrome cases treated with fetoscopic laser surgery according to gestational age (GA) at laser. [file UOG-67-461-s001.docx]

**Supplementary Table 1** - List of the participating centers

| **Participating Center** | **City and Country** | **Number of cases** |
| --- | --- | --- |
| St. George’s Hospital | London, UK | 200 |
| Royal Women’s Hospital | Melbourne, Australia | 11 |
| Chaim Sheba Medical Center, Tel Hashomer Hospital | Tel Aviv, Israel | 72 |
| University of Chieti | Chieti, Italy | 8 |
| Helen Schneider Hospital for Women, Rabin Medical Center, Petach Tikvah, Israel; Faculty of Medicine, Tel Aviv University, Tel Aviv, Israel. | Petah Tikva, Israel | 27 |
| Maternidade Dr Alfredo da Costa | Lisbon, Portugal | 34 |
| Spedali Civili di Brescia | Brescia, Italy | 47 |
| Copenhagen University Hospital Rigshospitalet | Copenhagen, Denmark | 91 |
| Policlinico Sant’Orsola | Bologna, Italy | 4 |
| Medical University of Vienna | Vienna, Austria | 27 |
| Leiden University Medical Center | Leiden, the Netherlands | 11 |
| University Medical Center Hamburg-Eppendorf | Hamburg, Germany | 19 |
| Hospital Universitari Vall d’Hebron, Universitat Autonoma de Barcelona | Barcelona, Spain | 54 |
| Università degli studi di Palermo | Palermo, Italy | 3 |
| La Fe University and Polytechnic Hospital | Valencia, Spain | 27 |
| Department of Gynecology and Obstetrics, Hospital das Clínicas, Ribeirão Preto Medical School, University of São Paulo | Ribeirão Preto, Brazil. | 2 |
| Burgerhospital, Frankfurt am Main | Frankfurt | 41 |

**Supplementary table 2a –** Complications within 7 days recorded according to gestational age (GA) and Quintero stage at diagnosis.

|  | **P-Prom** | **sIUD** | **Septostomy** | **Bleeding** | **Pregnancy Loss** |
| --- | --- | --- | --- | --- | --- |
| **GA ≤ 16+0 weeks (n=61)** | **18 (29.5)** | **14 (22.9)** | **2 (3.3)** | **3 (4.9)** | **4 (6.6)** |
| *Stage I (n=2)* | *1 (50)* | *1 (50)* | *0* | *0* | *0* |
| *Stage II (n=22)* | *4 (18.2)* | *3 (13.6)* | *1 (4.5)* | *0* | *0* |
| *Stage III (n=31)* | *12 (38.7)* | *8 (25.8)* | *1 (3)* | *3 (9.7)* | *4 (12.9)* |
| *Stage IV (n=6)* | *1 (16.7)* | *2 (33.3)* | *0* | *0* | *0* |
| **16+0 < GA ≤ 17+0 weeks (n=185)** | **22 (11.9)** | **35 (18.9)** | **5 (2.7)** | **7 (3.8)** | **16 (8.6)** |
| *Stage I (n=25)* | *2 (8)* | *5 (20)* | *1 (4)* | *0* | *3 (12)* |
| *Stage II (n=67)* | *9 (13.4)* | *9 (13.4)* | *2 (3.0)* | *2 (3.0)* | *4 (6.0)* |
| *Stage III (n=87)* | *11 (12.6)* | *20 (23.0)* | *2 (2.3)* | *5 (5.7)* | *8 (9.2)* |
| *Stage IV (n=6)* | *0* | *1 (16.7)* | *0* | *0* | *1 (16.7)* |
| **17+0 < GA ≤ 18+0 weeks (n=239)** | **27 (11.3)** | **43 (18.0)** | **8 (3.3)** | **7 (2.9)** | **14 (5.8)** |
| *Stage I (n=28)* | *1 (3.6)* | *3 (10.7)* | *0* | *0* | *0* |
| *Stage II (n=98)* | *12 (12.2)* | *18 (18.4)* | *5 (5.1)* | *2 (2.0)* | *7 (7.1)* |
| *Stage III (n=105)* | *13 (12.4)* | *20 (19.0)* | *3 (2.8)* | *5 (4.8)* | *6 (5.7)* |
| *Stage IV (n=8)* | *1 (12.5)* | *2 (25)* | *0* | *0* | *1 (12.5)* |

The data are presented as number (percentage) per raw

**Supplementary Table 2b –** Late complications recorded according to gestational age (GA) and Quintero stage at diagnosis.

|  | **post laser TAPS** | **recurrent TTTS** | **post laser sFGR** | **repeated laser** |
| --- | --- | --- | --- | --- |
| **GA ≤ 16+0 weeks (n=61)** | **5/54 (8.2)** | **1/54 (1.6)** | **7/50 (11.5)** | **0** |
| *Stage I (n=2)* | *0* | *0* | *0* | *0* |
| *Stage II (n=22)* | *2/19 (9.1)* | *0* | *3/19 (13.6)* | *0* |
| *Stage III (n=31)* | *3/28 (9.7)* | *1/28 (3.2)* | *4/27 (12.9)* | *0* |
| *Stage IV (n=6)* | *0* | *0* | *0* | *0* |
| **16+0 < GA ≤ 17+0 weeks (n=185)** | **12/163 (6.5)** | **7 (3.2)** | **19/157 (9.2)** | **2 (1.1)** |
| *Stage I (n=25)* | *0* | *3/21 (8)* | *1/21 (4)* | *1 (4)* |
| *Stage II (n=67)* | *9/59 (13.4)* | *3/60 (4.5)* | *7/60 (8.9)* | *0* |
| *Stage III (n=87)* | *3/78 (3.4)* | *1/78 (1.1)* | *10/73 (10.3)* | *1 (1.1)* |
| *Stage IV (n=6)* | *0* | *0* | *1/3 (16.7)* | *0* |
| **17+0 < GA ≤ 18+0 weeks (n=239)** | **14/200 (5.9)** | **13/201 (5.4)** | **20/203 (8.8)** | **4 (1.7)** |
| *Stage I (n=28)* | *4/23 (14.3)* | *0* | *4/23 (14.3)* | *0* |
| *Stage II (n=98)* | *2/76 (2)* | *5/77 (5.1)* | *3/77 (3.1)* | *0* |
| *Stage III (n=105)* | *8/96 (7.6)* | *8/96 (6.7)* | *12/98 (12.4)* | *3 (2.9)* |
| *Stage IV (n=8)* | *0* | *0* | *1/5 (12.5)* | *1 (12.5)* |

The data are presented as number (percentage) per raw

**Supplementary Table 3a** – Perinatal outcomes of cases of early TTTS diagnosed at GA ≤ 16+0 (n=61) and treated with laser according to Quintero stage at diagnosis and timing of treatment from diagnosis.

| **N=61** | **No survivors** | **Single survival** | **Dual survival** |
| --- | --- | --- | --- |
| **Stage I (n=2)** | **0** | **1 (50)** | **1 (50)** |
| *Laser ≤ 72 hours (n=0)* | */* | */* | */* |
| *Laser between 72 hours and 1 week(n=0)* | */* | */* | */* |
| *Laser > 1 week (n=2)* | *0* | *1 (50)* | *1 (50)* |
| **Stage II (n=22)** | **3 (13.6)** | **5 (22.7)** | **14 (63.6)** |
| *Laser ≤ 72 hours (n=8)* | *1 (14.3)* | *1 (14.3)* | *6 (75)* |
| *Laser between 72 hours and 1 week (n=3)* | *0* | *0* | *3(100)* |
| *Laser > 1 week (n=11)* | *2 (18.2)* | *4 (36.4)* | *5 (45.4)* |
| **Stage III (n=31)** | **13 (41.9)** | **8 (25.8)** | **10 (32.3)** |
| *Laser ≤ 72 hours(n=12)* | 6 (50) | 2 (16.7) | 4 (33.3) |
| *Laser between 72 hours and 1 week (n=5)* | 1 (20) | 2 (40) | 2 (40) |
| *Laser > 1 week (n=14)* | 6 (42.9) | 4 (28.6) | 4 (28.6) |
| **Stage IV (n=6)** | **2 (33.3)** | **1 (16.7)** | **3 (50)** |
| *Laser ≤ 72 hours (n=4)* | 1 (25) | 1 (25) | 2 (50) |
| *Laser between 72 hours and 1 week (n=1)* | / | / | 1 (100) |
| *Laser > 1 week (n=1)* | 1 (50) | 0 | 0 |

The data are presented as number (percentage) per raw

**Supplementary Table 3b** – Perinatal outcomes of cases of early TTTS diagnosed at 16+1 < GA ≤ 17+0 (n=184) and treated with laser according to Quintero stage at diagnosis and timing of treatment from diagnosis.

| **N=184** | **No survivors** | **Single survival** | **Dual survival** |
| --- | --- | --- | --- |
| **Stage I (n=24)^#^** | **4 (16.7)** | **7 (29.2)** | **13 (54.2)** |
| *Laser ≤ 72 hours (n=10)* | *2 (20)* | *3 (30)* | *5 (50)* |
| *Laser between 72 hours and 1 week(n=3)* | *1 (33.3)* | *0* | *2 (66.7)* |
| *Laser > 1 week (n=11)* | *1 (9.1)* | *4 (36.4)* | *6 (54.5)* |
| **Stage II (n=67)** | **12 (17.9)** | **19 (28.3)** | **36 (53.7)** |
| *Laser ≤ 72 hours (n=44)* | *10 (22.7)* | *12 (27.3)* | *22 (50)* |
| *Laser between 72 hours and 1 week (n=11)* | *2 (18.2)* | *3 (27.3)* | *6 (54.5)* |
| *Laser > 1 week (n=12)* | *0* | *4 (33.3)* | *8 (66.6)* |
| **Stage III (n=87)** | **21 (24.1)** | **19 (21.8)** | **47 (54)** |
| *Laser ≤ 72 hours(n=69)* | *19 (27.5)* | *15 (21.7)* | *35 (50.7)* |
| *Laser between 72 hours and 1 week (n=10)* | *0* | *3 (30)* | *7 (70)* |
| *Laser > 1 week (n=8)* | *2 (25)* | *1 (12.5)* | *5 (62.5)* |
| **Stage IV (n=6)** | **1 (16.7)** | **2 (33.3)** | **3 (50)** |
| *Laser ≤ 72 hours (n=5)* | 1 (20) | 2 (40) | 2 (40) |
| *Laser between 72 hours and 1 week (n=0)* | / | / | / |
| *Laser > 1 week (n=1)* | 0 | 0 | 1 (100) |

**^#^** One case of stage 1 had no info on timing and dual survival;

The data are presented as number (percentage) per raw

**Supplementary Table 3c** – Perinatal outcomes of cases of early TTTS diagnosed at 17+1 < GA ≤ 18+0 (n=234) and treated with laser according to Quintero stage at diagnosis and timing of treatment from diagnosis.

| **N=235** | **No survivors** | **Single survival** | **Dual survival** |
| --- | --- | --- | --- |
| **Stage I (n=27)^#^** | **3 (11.1)** | **4 (14.8)** | **20 (74.1)** |
| *Laser ≤ 72 hours (n=7)* | *0* | *1 (14.3)* | *6 (85.7)* |
| *Laser between 72 hours and 1 week(n=5)* | *0* | *2 (40)* | *3 (60)* |
| *Laser > 1 week (n=15)* | *3 (20)* | *1 (6.7)* | *11 (73.3)* |
| **Stage II (n= 96)*** | **26 (27.1)** | **22 (22.9)** | **48 (50)** |
| *Laser ≤ 72 hours (n=75)* | *25 (33.3)* | *20 (26.7)* | *30 (40)* |
| *Laser between 72 hours and 1 week (n=5)* | *1 (20)* | *0* | *4 (80)* |
| *Laser > 1 week (n=16)* | *0* | *2 (13.3)* | *14 (93.3)* |
| **Stage III (n=105)** | **24 (22.3)** | **31 (29.5)** | **50 (47.6)** |
| *Laser ≤ 72 hours(n=99)* | 24 (24.2) | 27 (27.3) | 48 (48.5) |
| *Laser between 72 hours and 1 week (n=0)* | / | / | / |
| *Laser > 1 week (n=6)* | 0 | 4 (66.7) | 2 (33.3) |
| **Stage IV (n=7)^§^** | **3 (42.9)** | **2 (28.6)** | **2 (28.6)** |
| *Laser ≤ 72 hours (n=5)* | 2 (40) | 2 (40) | 1 (20) |
| *Laser between 72 hours and 1 week (n=0)* | / | / | / |
| *Laser > 1 week (n=2)* | 1 (50) | 0 | 1 (50) |

**^#^** One case of stage I had no info on timing and dual survival; * Two cases of stage II had no info on timing and single and dual survival; **^§^**One case of stage IV had no info on timing and dual survival;

The data are presented as number (percentage) per raw

**Table S4** Comparison of rates of survival of at least one twin in Quintero Stages I-II and Stages III-IV twin-to-twin transfusion syndrome cases treated with fetoscopic laser surgery according to gestational age (GA) at laser

| Quintero Stage | GA at laser (weeks) | | | *P* |
| --- | --- | --- | --- | --- |
|  | ≤ 16 + 0 | 16 + 1 to 18 + 0 | > 18 + 0 |  |
| Stages I-II | *4/5 (80.0)* | *129/166 (77.7)* | *57/67 (85.1)* | 0.44 |
| Stages III-IV | *8/13 (61.5)* | *151/206 (73.3)* | *19/23 (82.6)* | 0.378 |

Data are given as *n/N* (%).
